# Cardiac Arrhythmias: Advances in Mechanisms, Diagnosis, and Treatment

**DOI:** 10.3390/life16060968

**Published:** 2026-06-09

**Authors:** Paschalis Karakasis, Antonios P. Antoniadis, Nikolaos Fragakis

**Affiliations:** Second Department of Cardiology, Hippokration General Hospital, Aristotle University of Thessaloniki, 54642 Thessaloniki, Greece; aantoniadis@gmail.com (A.P.A.); fragakis.nikos@googlemail.com (N.F.)

**Keywords:** cardiac arrhythmias, atrial fibrillation, arrhythmia substrate, risk stratification, structural remodeling, autonomic dysfunction, inflammation

## Abstract

Cardiac arrhythmias are increasingly recognized as dynamic clinical phenotypes arising from the interplay between myocardial substrate, systemic biology, and modifiable exposures rather than as isolated disorders of cardiac electrophysiology. Advances in the field have broadened the conceptual framework of arrhythmia medicine to include structural remodeling, inflammation, autonomic dysfunction, metabolic perturbation, endothelial injury, and aging-related vulnerability as central determinants of arrhythmic risk, progression, and treatment response. In parallel, diagnostic paradigms are evolving from rhythm classification alone toward multidimensional phenotyping that integrates clinical, physiological, and imaging-based markers to identify susceptibility earlier and with greater precision. These developments are also reshaping therapeutic strategy, supporting a shift from uniform treatment algorithms toward individualized care in which rhythm control, surveillance, risk-factor modification, anticoagulation, and antiarrhythmic drug selection are aligned with the underlying biological context. This more integrated view positions arrhythmias not simply as electrical events to be suppressed, but as manifestations of broader cardiovascular and systemic disease processes that require mechanistically informed and phenotype-directed management.

As research on cardiac arrhythmias moves beyond rhythm description toward mechanism-based interpretation and individualized care, the Special Issue “Advances in Cardiac Arrhythmias: Mechanisms, Diagnostics and Therapeutics” brings together contributions that reflect this broader evolution of the field. Cardiac arrhythmias are increasingly recognized not as isolated disturbances of impulse generation and conduction, but as clinical manifestations of a far broader and biologically active disease continuum. Structural remodeling, inflammation, autonomic dysfunction, metabolic stress, endothelial injury, aging-related vulnerability, and multimorbidity collectively shape arrhythmic susceptibility, modulate phenotypic expression, and influence therapeutic response [1]. Viewed through this lens, arrhythmia care is undergoing a conceptual shift: away from rhythm classification as the dominant organizing principle and toward a more integrated framework grounded in substrate, mechanism, and clinical context [2,3]. Across a range of contemporary settings—including AF with heart failure, diabetes, chronic kidney disease, obstructive sleep apnea, cardio-oncology, post-COVID syndrome, and post-ablation atrial disease—recent work increasingly converges on the same central message: progress in the field will depend on more precise phenotyping and more individualized, biology-informed management [4].

One review included in the Special Issue examines arrhythmia-induced cardiomyopathy in AF [5]. The article reframes AF not merely as an associated finding in patients with ventricular dysfunction, but in selected cases as a direct and potentially reversible driver of myocardial impairment. Importantly, it moves beyond the traditional rate-centric explanation and instead emphasizes calcium-handling abnormalities, oxidative and metabolic stress, fibro-inflammatory signaling [1], and atrial–ventricular crosstalk as central mechanisms of disease. The review also highlights the increasingly persuasive case for early rhythm control, especially catheter ablation [6], as a disease-modifying rather than merely symptom-relieving intervention [5]. In doing so, it captures a defining development in contemporary electrophysiology: the recognition that reverse remodeling may be achievable when arrhythmic burden is reduced early and substantially [7]. At the same time, the paper appropriately underscores persistent uncertainties, including the absence of standardized diagnostic criteria, the lack of validated burden thresholds for recovery, and unresolved questions regarding therapeutic sequencing and de-escalation after apparent improvement [5].

A second central theme of the Special Issue is that arrhythmic risk often becomes detectable before clinical events occur, provided that we measure the right physiological or imaging signals. The review on heart rate variability in type 2 diabetes is illustrative in this respect [8]. By synthesizing evidence linking cardiac autonomic neuropathy to reduced HRV and heightened arrhythmogenic vulnerability, it brings needed attention to the role of autonomic dysfunction as an upstream driver of both atrial and ventricular rhythm instability. The importance of this contribution lies not only in its mechanistic relevance but also in its practicality. HRV is inexpensive, non-invasive, and widely accessible, yet remains underused in routine cardiovascular risk assessment. The review argues that reductions in conventional and nonlinear HRV indices may identify patients with diabetes who harbor subclinical autonomic impairment and therefore heightened rhythm risk before more overt cardiovascular manifestations emerge [8]. In an era increasingly defined by precision medicine, this is a valuable reminder that precision does not necessarily depend on technological complexity; it may also depend on the more thoughtful use of established physiological tools.

The review addressing AF in chronic kidney disease extends this substrate-centered perspective into one of the most difficult therapeutic domains in contemporary cardiovascular medicine [9]. AF and CKD represent a particularly unstable interface in which thrombosis and bleeding coexist, often driven by shared biological disturbances rather than by distinct and opposing pathways. By focusing on systemic inflammation, endothelial dysfunction, platelet dysregulation, oxidative stress, and altered coagulation, the review makes a compelling case that conventional anticoagulation paradigms—still largely anchored to creatinine clearance—capture pharmacokinetic safety but insufficiently represent the underlying biology of thrombohemorrhagic risk [9]. This is an important contribution because it challenges the field to rethink risk stratification in AF with CKD as something more dynamic and biologically heterogeneous than current static scoring models allow. In that sense, the review does more than summarize mechanisms; it proposes a reframing of anticoagulation decision-making itself.

The original investigations in this Special Issue reinforce the same broader message: arrhythmias arise within identifiable clinical and biological contexts, and meaningful prevention depends on recognizing those contexts early. The study of hospitalized survivors of severe COVID-19 found a high prevalence of arrhythmias during long COVID [10], with AF emerging as the most frequent rhythm disorder and older age and longer hospitalization associated with increased risk. Although limited by its cross-sectional design and modest sample size, the study is important because it supports the notion that post-viral syndromes may sustain a persistent arrhythmogenic substrate, potentially through inflammatory activation, myocardial injury, fibrosis, autonomic imbalance, or chronic active myocarditis [10]. As post-COVID cardiovascular medicine continues to mature, this work helps frame arrhythmia surveillance as an important component of longer-term follow-up in selected high-risk populations.

A similarly forward-looking perspective emerges from the pilot study of patients treated with ibrutinib [11]. In cardio-oncology, AF is not simply a cardiovascular complication; it may alter oncologic continuity of care and force difficult therapeutic trade-offs. This study suggests that such risk may be foreseeable before treatment begins [11]. Patients who later developed AF had lower baseline peak atrial contraction strain, while left atrial functional parameters remained broadly stable during early follow-up. These findings support a substrate–trigger model in which ibrutinib acts less as a uniformly toxic insult and more as a precipitant in patients with pre-existing atrial vulnerability [11]. This distinction is clinically important. It shifts the focus from reactive toxicity management to proactive phenotyping and structured surveillance, and it supports a role for advanced echocardiographic markers—particularly left atrial contractile function—in pre-treatment cardio-oncology assessment.

The prospective cohort evaluating CPAP adherence in patients with severe obstructive sleep apnea and paroxysmal AF [12] offers an equally relevant, though distinct, lesson. The study found that greater objectively measured nightly CPAP exposure was independently associated with delayed AF recurrence when analyzed as a continuous variable, whereas the conventional dichotomous adherence threshold of 4 h/night was not independently predictive [12]. This is more than a methodological detail. It suggests that the arrhythmic benefit of OSA treatment may be graded rather than binary, and that clinically meaningful exposure–response relationships can be obscured when treatment adherence is reduced to a simplistic cutoff. The study therefore contributes to a more quantitative and mechanistically plausible model of risk-factor modification in AF, one that links objectively measured therapeutic exposure to rhythm outcome.

At the opposite end of the technological spectrum, the study examining diagonal earlobe crease after cavotricuspid isthmus ablation for typical atrial flutter reminds us that useful arrhythmic risk markers are not always sophisticated [13]. In this cohort, the presence of an earlobe crease was independently associated with new-onset AF after ablation, suggesting that a simple physical sign may reflect broader vascular, inflammatory, and aging-related processes relevant to atrial remodeling [13]. The significance of this paper lies less in the marker itself than in what it represents conceptually: a willingness to revisit overlooked clinical phenotypes as potential tools for risk enrichment. In an era dominated by advanced imaging and digital monitoring, the study is a timely reminder that careful bedside observation still has value when integrated into a mechanistically informed framework.

In another contribution to the Special Issue we discuss flecainide use in selected patients with structural heart disease [14]. Although distinct in focus from the risk-prediction and mechanistic papers, it addresses an equally important frontier in arrhythmia therapeutics: the reassessment of long-standing treatment dogma in the light of contemporary substrate characterization. The review argues that the historic caution surrounding flecainide, driven largely by the CAST experience, has often been extrapolated beyond the specific ischemic and post-infarction phenotype actually studied. It suggests that in carefully selected patients—such as those with stable coronary artery disease without active ischemia, preserved ventricular function, arrhythmogenic right ventricular cardiomyopathy, or premature ventricular complex-induced cardiomyopathy—flecainide may offer meaningful antiarrhythmic benefit without a uniform signal of excess proarrhythmia or mortality. Rather than promoting indiscriminate liberalization, the paper advocates a mechanistically informed and individualized reappraisal of therapy, supported by advances in cardiac imaging, ischemia assessment, and phenotypic risk stratification.

Taken together, the papers in this Special Issue indicate that cardiac arrhythmia research is moving along three major axes. The first is a deepening mechanistic understanding of arrhythmia as a manifestation of systemic and myocardial substrate rather than a purely electrical phenomenon. The second is the expansion of precision diagnostics, including imaging, physiological monitoring, exposure-based metrics, and even simple clinical phenotypes that help identify risk before overt clinical deterioration. The third is the gradual emergence of individualized therapeutics, in which rhythm control, risk-factor modification, anticoagulation, surveillance, and antiarrhythmic drug selection are increasingly tailored to biologic context rather than applied according to rigid, one-size-fits-all rules.

The Special Issue also reflects the limitations of the field at its current stage. Several original studies are necessarily exploratory, constrained by modest sample size, observational design, single-center recruitment, or limited follow-up. The reviews, though conceptually rich, also expose the relative scarcity of prospective validation for many promising pathways and markers. Yet this should not be viewed as a weakness. Rather, it accurately reflects a field in transition: one in which mechanistic understanding has outpaced definitive clinical algorithm development. The central challenge moving forward will be integration—embedding biological, imaging, physiological, and clinical signals into decision pathways that are rigorous, scalable, and prospectively testable.

Finally, cardiac arrhythmia research is entering a more mature and clinically consequential phase. The field is moving beyond reductionist models that treat rhythm disorders as isolated electrical events and toward a more integrated framework in which substrate, systemic biology, comorbidity burden, and treatment exposure are understood as co-determinants of arrhythmic risk and therapeutic response. This evolution carries important implications for practice. Future progress will depend on combining mechanistic insight with clinically scalable tools, refining phenotypic classification, and testing management strategies that are tailored not only to the arrhythmia itself but also to the biological context in which it arises. In that sense, the future of arrhythmia medicine will be defined less by uniform algorithms than by increasingly precise, multidimensional, and individualized care.

## Data Availability

All data generated in this research is included within the article.
